# Integrative omics and field studies reveal the efficacy of PlantGuard, a next-generation biostimulant

**DOI:** 10.3389/fpls.2026.1918122

**Published:** 2026-09-30

**Authors:** Walter Stünkel, Tigran Davtyan, Anna Prazyan, Haig Babikian, Rubiyanto W. Haliman, Muji Winarno, Hendri Laiman, Benjamin Jiaravanon, Yusef Babikyan

**Affiliations:** 1 Rhea Natural Sciences Pte. Ltd., Singapore, Singapore; 2 PT. Central Proteina Prima Tbk, Jakarta, Indonesia

**Keywords:** biostimulant, essential oils, multi-omics, plant stress response, transcriptomics

## Abstract

This study presents the development and evaluation of a novel, natural plant oil-based biostimulant called PlantGuard, formulated from a blend of essential oil components and emulsifiers designed to enhance crop performance under field and controlled-environment hydroponics conditions. The biostimulant was applied to horticultural vegetables and crops such as tomato (*Solanum lycopersicum*) and shallot *(Allium cepa* var. *aggregatum)*, as well as rice (*Oryza sativa*) across multiple trial sites in Indonesia over two growing seasons. Treated plots demonstrated statistically significant improvements in flowering synchrony, fruit set, biomass accumulation, and overall yield, with increases ranging from 15% to 32% compared to untreated controls. We also studied the effect of PlantGuard on tomatoes and kale (*Brassica oleracea* var. *sabellica)*, grown under hydroponic conditions and recorded an almost 3-fold increase in total yield. To characterize biological responses associated with PlantGuard, a comprehensive molecular analysis was conducted comprising RNA sequencing, quantitative proteomics, and untargeted metabolomics. RNA-sequencing profiling revealed differential expression of genes associated with photosynthesis, nutrient transport, hormonal signaling, including jasmonic acid- and auxin-related responses, and defense processes. Proteomic analyses confirmed the enhanced expression of stress-related proteins, antioxidant enzymes, and metabolic regulators. Metabolomic fingerprinting identified elevated levels of phenylpropanoids, flavonoids, and lipid-derived signaling molecules associated with abiotic stress mitigation and plant growth promotion. Collectively, these multi-omics findings validate the functional activity of PlantGuard at the molecular level and support its application as a sustainable input for enhancing crop and vegetable productivity by increasing plant growth and resilience in diverse agricultural systems.

## 1 Introduction

The escalating pressures of global food demand, diminishing arable land, and the unpredictable impacts of climate change have put unprecedented strain on agricultural systems, necessitating innovative solutions to sustainably intensify crop production. In this context, plant biostimulants—defined as substances or microorganisms that enhance plant growth, stress tolerance, and nutrient efficiency without supplying primary nutrients—have emerged as vital tools in modern agronomy (du Jardin, 2015; Calvo et al., 2014). While traditional biostimulants, such as humic and fulvic acids, protein hydrolysates, seaweed extracts, and microbial inoculants, have shown considerable promise in improving crop performance, their outcomes can be inconsistent under variable field conditions (Yakhin et al., 2017; Rouphael and Colla, 2020). This variability has spurred interest in diversifying biostimulant sources and mechanisms of action.

Essential oils (EOs) from aromatic plants have attracted increasing interest as plant biostimulants. Their complex mixtures of terpenoids, phenylpropanoids, and other volatile compounds not only possess antimicrobial and insect-repellent properties but can also influence plant physiology and stress signaling (Kourdova et al., 2025). A practical challenge associated with EO-based formulations is their hydrophobicity, which can limit dispersion and foliar delivery. Non-ionic emulsifiers such as polysorbates (e.g., Tween 20 and Tween 80) can improve the dispersion, adhesion, and stability of EOs in aqueous spray formulations and facilitate more uniform foliar coverage (Kourdova et al., 2025). Field studies with formulations combining plant extracts and humic acids have also demonstrated improvements in biomass and essential-oil quality in lavender (El-Hefny and Hussien, 2025). Nevertheless, compared with more established classes of biostimulants, EO-based formulations remain relatively poorly characterized with respect to reproducible agronomic performance across different crops and, particularly, their underlying molecular effects.

Against this background, we developed PlantGuard, an essential-oil-based biostimulant formulated from plant-derived volatile components and non-ionic emulsifiers. To evaluate its agronomic activity across complementary cultivation systems, PlantGuard was investigated in field trials involving tomato, shallot and rice, as well as under controlled hydroponic conditions using kale and tomato. To complement the field studies, hydroponic experiments were included to examine PlantGuard-associated growth responses under more controlled cultivation conditions.

Hydroponics is a soilless cultivation approach in which plants are supplied with nutrients through aqueous solutions and is increasingly used for controlled and urban agriculture. Hydroponic systems can substantially reduce water consumption compared with conventional soil-based cultivation while enabling year-round production (Hochmuth and Hochmuth, 2003). Kale is particularly suitable for hydroponic production because of its compact growth, short crop cycle and high nutritional value. Recent studies have also demonstrated the economic feasibility of hydroponic production systems incorporating kale and other leafy vegetables (Ichdayati et al., 2025).

Beyond evaluating agronomic performance, a further objective of this study was to investigate biological responses associated with PlantGuard treatment. Tomato was used as the molecular model, and transcriptomic, quantitative proteomic and untargeted metabolomic analyses were applied to identify treatment-associated changes in gene expression, protein abundance and metabolic pathways. Thus, a principal feature of this study is the combination of multi-crop agronomic evaluation across field and controlled cultivation systems with multi-omics characterization of an essential-oil-based biostimulant, thereby examining observed plant-performance responses alongside candidate molecular and physiological processes associated with PlantGuard treatment.

## 2 Materials and methods

### 2.1 Agricultural studies

PlantGuard was applied to all field crops by foliar spraying. Treatment solutions were prepared at the PlantGuard concentrations specified for the respective experiments and applied using a spray volume equivalent to 500 L/ha. Thus, for example, application at 0.6 mL/L corresponded to 300 mL PlantGuard/ha. PlantGuard consisted of 70% (v/v) oil components and 30% (v/v) Tween 80, the latter serving as the non-ionic surfactant.

Field trials were conducted using randomized block designs adapted to the respective experiments. A representative randomized block layout comprised five treatments with four replications. Each experimental unit (replicate plot) covered 5 m², corresponding to 20 m² per treatment and a total trial area of 100 m².

Representative baseline soil and water characteristics across the field trial sites were determined from average measurements obtained under the cultivation conditions used in these studies. Soil pH ranged from approximately 5.5 to 6.0, while irrigation-water pH was approximately 5.0. A representative soil composition included 2.52% organic carbon and 0.22% total N. Available P_2_O_5_ was 12.03 mg/kg, while potential P_2_O_5_ was 23.43 mg/100 g. Available K_2_O was 56.98 mg/kg, while potential K_2_O was 20.31 mg/100 g.

#### 2.1.1 Tomato field trial

The tomato field trial was conducted in Cijembar Village, Pangalengan District, Bandung Regency, Indonesia, using the TM Blazer tomato variety. The study was performed in field plots measuring 3.0 m × 1.7 m per replication, corresponding to 5.1 m² per plot. Each treatment consisted of three replications, giving a total treatment area of approximately 15 m² (15.3 m² rounded to 15 m²), within an overall trial land unit of 107 m². The experimental population comprised 30 plants per treatment. Treatments were applied at 10-day intervals for a total of three applications at 40, 50, and 60 days after planting (DAP). The trial followed a randomized block design with seven treatments and three replications. Agronomic observations included number of flowers, number of fruits, and harvest results. The objective of the study was to compare the efficacy of PlantGuard products with competitor products under cultivation practices representative of tomato farmers in the Pangalengan area.

#### 2.1.2 Shallot field trial

The shallot field trial was conducted in Selorejo Village, Bagor District, Nganjuk Regency, Indonesia, using the Tajuk variety (Thailand–Nganjuk). The study was performed in field plots measuring 1.6 m × 3.0 m per replication, corresponding to 4.8 m² per plot. Each treatment consisted of three replications, resulting in a total treatment area of 14.4 m², within an overall trial land unit of 72 m² (comprising three replications across five treatments). Treatments were applied at 10-day intervals for a total of four applications at 10, 20, 30, and 40 days after planting (DAP), reflecting standard farmer practices. The trial followed a randomized block design with five treatments and three replications. Agronomic observations included plant height, number of bulbs, bulb diameter, and final harvest results. The objective of the study was to determine differences in plant performance between PlantGuard treatments, a commercial comparator, and an untreated control under practical field conditions.

#### 2.1.3 Rice field trials

Rice field trials were conducted at multiple locations in Karawang, West Java, Indonesia, over a cultivation period extending from early January to the end of April. The study design incorporated several trial units to evaluate different application frequencies of PlantGuard under practical farming conditions. In units 1–4, treatments were applied three times at 15, 30, and 60 days after planting (DAP). Units 5–8 received five applications at 15, 30, 45, 60, and 75 DAP, representing a more intensive treatment regime. An additional unit (unit 9) followed a modified schedule with three applications at 30, 45, and 60 DAP. All application timings were aligned with standard farmer practices in the Karawang region. Across all units, PlantGuard treatments were evaluated under field conditions representative of local rice cultivation, with comparisons made to untreated controls and, where applicable, commercial reference products. The objective of the study was to assess the performance of PlantGuard under different application schedules and to determine its efficacy in improving rice yield under real-world agronomic conditions.

#### 2.1.4 Hydroponics studies

Kale and tomato were grown in a Vertical Aeroponics System (VAS) located at the facility of the Singapore-headquartered company Tomato Town (https://thetomatotown.com). In this specific form of hydroponics plant roots are suspended in the air and misted with a nutrient-rich solution, comprising a method of growing plants without soil using a water-based nutrient delivery system.

An additional hydroponic experiment was conducted with tomato to assess whether the PlantGuard-associated growth response in 4 weeks old seedlings could be distinguished from a potential effect of the Tween 80-containing carrier. Six individual plants were used as biological replicates in each group. Treatment plants received PlantGuard at 0.6 mL/L, while control plants received a matched Tween 80/water carrier solution containing Tween 80 at the same final concentration present in the diluted PlantGuard treatment (0.18 mL/L). Stem length was measured for each plant and compared between groups using an unpaired two-tailed t-test with Welch’s correction.

### 2.2 Bioanalytics of PlantGuard oil composition

The GC–MS analysis of the raw oil mixture (blend of Eucalyptus, Lavender, Fennel and Jasmine) was performed on a Thermo Scientific GC gas chromatograph, coupled with a single quadrupole mass spectrometer. The GC was equipped with a fused silica capillary column TG-17MS, 30mx0.25mm, 0.25mcm, Helium was used as carrier gas at flow rate of 1.2 mL/min (split), and the injector temperature was 280 °C. The oven program started with an initial temperature of 40 °C held for 5 min, then the temperature was heated at 5 °C/min to 250 °C and held for 5 min. For single quadrupole MS detection, an election ionization system, with ionization energy of 70 eV was used. The sample (0.1 g) or alpha-terpineol, gamma-terpinene, eucalyptol standard samples (0.01 g) were diluted in 20 mL hexane, mixed well and subjected to the GS-MS analysis. The identification of the compounds was carried out by comparing retention indices and mass spectra with those obtained under identical experimental conditions. Data acquisition and processing were performed using a Thermo Scientific GC–MS software (e.g., Xcalibur) with deconvolution tools for accurate spectral interpretation. Mass spectra were matched against commercially available libraries, including the Wiley Registry of Mass Spectral Data (4th Ed.) and the NIST/EPA/NIH Mass Spectral Library (2012). For quantification of main compounds concentration in oil corresponding calibration curves have been used after running standard compounds diluted samples by GS-MS.

### 2.3 Sample preparation and RNA-sequencing

Tomato plants were grown in soil and treated by weekly foliage spray with either PlantGuard at a concentration of 0.6mL/L (treatment group). Untreated control plants received water only and an emulsifier-only control was not included in the RNA-seq experiment.

The RNA-seq experiment comprised five independent biological replicates per treatment, with each replicate representing one individual plant. Plants were randomly allocated to the control and PlantGuard treatment groups and cultivation positions were randomized to minimize potential positional effects. All five biological replicates per treatment were generated within a single experimental run. Accordingly, no experimental-run batch factor was included in the differential-expression analysis. The RNA-seq experiment comprised five independent biological replicates per treatment, with each replicate representing one individual plant.

PlantGuard treatment was initiated during the early seedling stage and applied at approximately weekly intervals. Plants used for RNA-seq had received two PlantGuard applications by 14 days of age, with the final application administered 24 h before tissue harvesting. PlantGuard was applied as a foliar spray using approximately three sprays per plant, sufficient to uniformly wet the foliage without excessive runoff, while control plants received water using the same application procedure.

Five untreated control plants (C1–C5) and five PlantGuard-treated plants (T1–T5) were used as independent biological replicates. For each replicate, the stem together with approximately three leaves was collected from one individual 14-day-old tomato plant. Leaf and stem tissues from each individual plant were pooled prior to RNA extraction, and the same sampling procedure was applied to all control and treated plants to standardize tissue composition across samples. The plant material was collected in ice-cold 96% ethanol kept on dry ice (for subsequent RNA-sequencing analysis) or immediately snap frozen in a dry ice-ethanol slurry (for subsequent proteomics and metabolomics analysis).

All RNA-sequencing studies were carried out by Novogene at their Singapore facility, where the extraction of total RNA and quality control were performed prior to library preparation. RNA quality was assessed according to the provider’s quality-control requirements for plant RNA used for directional mRNA library preparation, including assessment of RNA purity and integrity. Novogene’s documented requirements specify OD260/280 ≥2.0 and OD260/230 ≥2.0 by NanoDrop and RIN ≥5.8 by Agilent 2100, with no evidence of degradation or contamination. Samples passing quality control were advanced to library preparation.

For library construction, five untreated control plants (C1–C5) and five PlantGuard-treated plants (T1–T5) were used as independent biological replicates (n = 5 per group), with each biological replicate representing leaf and stem tissue collected from one individual 14-day-old tomato plant. Messenger RNA was purified from total RNA using poly-T oligo-attached magnetic beads. Directional mRNA libraries were prepared using the Fast RNA-seq Lib Prep Kit V2 (RK20306; ABclonal). Following mRNA fragmentation, first-strand cDNA was synthesized using random hexamer primers, followed by second-strand cDNA synthesis using dUTP to preserve library directionality. Libraries underwent end repair, A-tailing, adapter ligation, size selection, USER enzyme digestion, amplification, and purification. Library concentration and size distribution were assessed using Qubit, real-time PCR, and Bioanalyzer analysis. Libraries were sequenced on an Illumina NovaSeq 6000 platform using 150-bp paired-end sequencing (PE150). Sequencing generated approximately 79.6–103.2 million raw reads per sample (approximately 39.8–51.6 million paired-end read pairs), of which approximately 77.9–101.0 million clean reads per sample were retained following quality filtering.

Raw sequencing reads were processed using fastp v0.23.1 for quality filtering. Reads containing adapter contamination, reads containing more than 10% uncertain bases, and reads in which more than 50% of bases had a Phred quality score below 5 were removed.

The resulting clean reads were mapped to the *Solanum lycopersicum* SL3.0 reference genome (Ensembl Plants SL3.0, GCA_000188115.3) using HISAT2 v2.0.5. Gene-level read counts were obtained using featureCounts v1.5.0-p3.

Differential gene expression between PlantGuard-treated and untreated control samples was analyzed using DESeq2 v1.20.0, based on the negative binomial distribution. Untreated control samples were specified as the reference level; consequently, positive log_2_ fold-change values indicate higher expression in PlantGuard-treated samples relative to controls, whereas negative values indicate lower expression. p-values were adjusted for multiple testing using the Benjamini-Hochberg procedure (Benjamini and Hochberg, 1995), and genes with |log_2_(fold change)| ≥ 1 and an adjusted p-value (padj) ≤ 0.05 were considered differentially expressed.

Functional enrichment was performed using Gene Ontology (GO) and Kyoto Encyclopedia of Genes and Genomes (KEGG) annotations implemented through clusterProfiler v3.8.1, using *Solanum lycopersicum* SL3.0-associated gene identifiers and the whole-genome gene set as the background universe. GO terms and KEGG pathways with adjusted p-values (padj) ≤ 0.05 were considered significantly enriched. Enrichment analyses were performed on the combined set of significant DEGs. Upregulated and downregulated DEGs were not analyzed separately. Consequently, enrichment of a GO term or KEGG pathway was interpreted as over-representation among the DEGs rather than as evidence of pathway activation or suppression.

The RNA sequencing datasets generated and analyzed during the current study have been deposited in the NCBI Gene Expression Omnibus (GEO) under accession number GSE336220. The dataset will be publicly available upon publication of the manuscript or on the scheduled GEO release date.

### 2.4 DIA quantitative proteomics analysis

Proteomic and metabolomic analyses were conducted using material from 4-week-old tomato plants and these datasets therefore represent complementary analyses at different developmental stages rather than temporally matched multi-omics measurements.

Quantitative proteomics was carried out via Data-independent acquisition (DIA) at Novogene’s Singapore facility. The method divides the entire scanning range of mass spectrometry into several windows based on mass-to-charge ratio (m/z). Then, it fragments and detects all precursor ions in each window, collecting fragment ion information of all precursor ions for protein identification and quantification. Currently, popular proteomics research methods, such as iTRAQ/TMT, Label-free, and SILAC, use data-dependent acquisition (DDA). In DDA, the primary mass spectrometry performs a full scan to collect precursor ion signals, generating a primary spectrum. The secondary mass spectrometry sequentially selects the top 10/20/40 peaks in the primary spectrum based on signal intensity for fragmentation and collects corresponding fragment ion information. An Orbitrap Astral high-resolution mass spectrometer was employed which includes three mass analyzers: a quadrupole mass analyzer for selecting precursor ions, an Orbitrap mass analyzer for collecting high dynamic range HRAM spectra, and a new Astral mass analyzer capable of collecting high dynamic range HRAM spectra with high sensitivity and speeds up to 200Hz. The unique advantages of these mass analyzers combine to maximize the performance and usability of the solution. Proteins were extracted from 6 control and 6 treatment samples (stems and leaves), followed by quantification, detection, enzymatic digestion, desalting, fractionation, and mass spectrometry analysis. To ensure the accuracy and reliability of sequencing data from the outset, each experimental step at Novogene is rigorously controlled, fundamentally ensuring the production of high-quality data. Raw DIA mass spectrometry files were processed with DIA-NN software to generate peptide and protein identification and quantification data, followed by rigorous quality control (QC) reporting to ensure reproducibility and accuracy. Protein abundance data were subjected to quantitative analyses, including principal component analysis (PCA) and repeatability assessments across biological replicates. Differential protein analysis was then carried out, with statistical filtering to identify significantly up- or downregulated proteins, and further explored using volcano plots, cluster analysis, and k-means clustering. Functional annotation of proteins included Gene Ontology (GO), Clusters of Orthologous Groups (COG), Kyoto Encyclopedia of Genes and Genomes (KEGG), and InterPro (IPR) annotation, as well as subcellular localization and transcription factor predictions. Differentially abundant proteins were also analyzed through protein–protein interaction (PPI) networks using Cytoscape, and enrichment analyses (GO, KEGG, IPR, subcellular) were performed to interpret affected pathways. This multi-layered approach enabled robust identification and biological interpretation of PlantGuard–induced proteomic changes.

### 2.5 Untargeted metabolomics analysis

Liquid chromatography-mass spectrometry (LC-MS) was used to conduct untargeted metabolomics from 6 control and 6 treatment samples (leaves and stems). All experimental work was carried out at Novogene’s Singapore facility and included sample metabolite extraction, LC-MS/MS detection and data analysis. Plant samples were first collected and subjected to metabolite extraction and pretreatment to ensure reproducible recovery of low-molecular-weight compounds. Extracts were analyzed by LC-MS/MS, with both experimental and quality control (QC) samples included to monitor instrument performance and data stability. Detected features were then processed for qualitative and quantitative analysis, with metabolite identification performed against multiple public and proprietary databases. Finally, bioinformatics (BI) analysis integrated quantitative and qualitative data to generate pathway-level insights, including enrichment analysis and biological interpretation of differentially abundant metabolites between PlantGuard–treated and control groups. Raw LC-MS metabolomics data were processed through a multi-step bioinformatics workflow to ensure high-quality metabolite identification and interpretation. Following data pretreatment and database searching, metabolites were annotated using multiple resources including KEGG, HMDB, and LIPID MAPS. Rigorous data quality control (QC) was performed, including PCA analysis to evaluate sample clustering and reproducibility. Differential metabolite screening was conducted using PCA, PLS-DA, and volcano plot analyses to identify significantly altered metabolites between PlantGuard–treated and control groups. Identified metabolites were subjected to differential analysis, incorporating hierarchical clustering, correlation analysis, and Z-score diagrams to explore group-specific patterns. Biological interpretation was carried out through KEGG enrichment analysis, including bubble and pathway diagrams, to map metabolites to functional pathways. Additional validation included ROC analysis of differential metabolites, ensuring robustness of selected biomarkers. Finally, results were integrated into a comprehensive final report summarizing quantitative changes and pathway-level insights.

### 2.6 Statistical methods

All experiments were conducted using independent biological replicates (n = 24 per treatment group unless otherwise stated). Data are presented as mean ± standard error of the mean (SEM). Statistical analyses were performed to evaluate differences between treatment groups and the control, as well as between PlantGuard treatments and a competitor product. For comparisons involving more than two groups, a one-way analysis of variance (ANOVA) was applied, followed by appropriate *post hoc* multiple comparison testing to identify significant pairwise differences. In cases where direct comparisons between two groups were required, a two-tailed Student’s t-test was used. Normality and homogeneity of variance were assumed based on the experimental design and sample size. Statistical significance was defined at thresholds of p ≤ 0.05 and p ≤ 0.01. These significance levels are indicated in figures and tables using asterisks (p ≤ 0.05; p ≤ 0.01). All analyses were conducted using standard statistical software.

## 3 Results

GC–MS analysis of the PlantGuard oil formulation identified a diverse mixture of volatile organic compounds (VOCs), including monoterpenes, benzenoids, esters, alcohols, and ketones (**Table 1**). Major detected compounds included linalool, eucalyptol (1,8-cineole), α-terpineol, γ-terpinene, benzaldehyde, and benzyl acetate. These data characterize the principal volatile constituents of the PlantGuard formulation used in the subsequent efficacy and molecular studies.

**Table 1:** Major volatile compounds identified in PlantGuard by GC–MS analysis.

Rt	Name	Area %
18.89	Menthol	22.83
14.57	ç-Terpinene	16.07
20.16	à-Terpineol	14.75
31.22	2-Butanone, 4-(4-methoxyphenyl)-	14.51
13.94	Eucalyptol	5.4
16.84	1-Propanol, 2-(2-hydroxypropoxy)-	5.32
14.66	Benzaldehyde	2.97
16.37	Linalool	2.76
14.96	7-Octen-2-ol, 2,6-dimethyl-	1.63
15.43	Ethanol, 2-(2-ethoxyethoxy)-	1.46
8.6	1-Butanol, 3-methyl-, acetate	1.38
21.18	Acetic acid, phenylmethyl ester	1.07

The table lists the most abundant constituents of PlantGuard oil, including their retention times (Rt) and relative peak areas (% of total). Cyclohexanol, 5-methyl-2-(1-methylethyl) was the dominant compound (22.83%), followed by γ-terpinene (16.07%), α-terpineol (14.75%), and 2-butanone, 4-(4-methoxyphenyl) (14.51%). Other detected volatiles included eucalyptol, benzaldehyde, and linalool, as well as several alcohols, esters, and phenolic derivatives. These compounds are known to contribute to plant defense, stress tolerance, and potential signaling activities, providing a chemical basis for the observed bioactivity of PlantGuard.

### 3.1 Efficacy of PlantGuard in field studies with common vegetables

A field study was conducted in the highland agricultural region of Pangalengan District, Bandung Regency, West Java Province, Indonesia, to evaluate the efficacy of PlantGuard (PG) on tomato productivity under real-world farming conditions. The trial tested five different doses of a 4000ppm stock solution of PG (0.24-1.5mL/L), alongside an untreated control, using a randomized block design with standard agronomic practices. Among all treatments, PG at 0.24 mL/L produced the highest fruit count, averaging 28.7 ± 2.80 fruits per plant, and achieved the greatest total harvest weight at 18.31 ± 0.42 kg per plot compared to the control which produced 13.81 ± 0.35 kg (**Table 2**). This corresponds to a 32.6% increase in the yield for the PG-treated tomato group. PG concentrations of 0.3 mL/L and 0.6 mL/L also showed strong performance, resulting in significantly higher yields than the control. The untreated control yielded the lowest fruit count and one of the lowest harvest weights. Among the concentrations evaluated, PG at 0.24 mL/L produced the highest tomato yield under the environmental conditions of Pangalengan.

**Table 2:** Effect of PlantGuard (PG) on tomato yield in field trials conducted in Indonesia.

Treatment	N Data	Number of fruits (pcs)	Number of flowers (pcs)	Total harvest (kg)
Control	3	21.3 ± 2.01	3.1 ± 0.42	13.81 ± 0.35
Competitor 2 mL/L	3	22.3 ± 3.95	3.9 ± 1.62	14.33 ± 0.68
PG 0.24 mL/L	3	28.7 ± 2.80*	3.5 ± 1.33	18.31 ± 0.42**
PG 0.30 mL/L	3	24.2 ± 1.78	3.5 ± 2.34	17.34 ± 0.85
PG 0.60 mL/L	3	23.0 ± 4.80	3.4 ± 1.64	13.12 ± 0.27
PG 1.00 mL/L	3	23.1 ± 0.50	4.3 ± 1.14	16.85 ± 1.67
PG 1.50 mL/L	3	25.5 ± 2.91	2.2 ± 0.80	12.54 ± 0.19
p-value		*≤ 0.05		**≤ 0.01

Tomato plants were treated with different concentrations of PlantGuard (PG) and compared with untreated controls and a competitor product (2 mL/L). Data are shown as mean ± standard error (n = 3). PG treatment at 0.24 mL/L resulted in a significant increase in the number of fruits (28.7 ± 2.80, p = 0.03) compared with control and competitor treatments. The total harvest weight was also significantly improved in the PG 0.24 mL/L group (18.31 ± 0.42 kg, p = 0.01), representing the highest yield among all treatments. Other PG concentrations showed variable effects, with PG 1.0 mL/L also producing a relatively high yield (16.85 ± 1.67 kg). These results indicate that low-dose PG application can substantially enhance fruit production and overall yield in tomato cultivation under field conditions. Statistical significance was defined as *p **≤** 0.05 and **p **≤** 0.01.

We therefore carried out a small field study in Selorejo Village, Bagor District, Nganjuk Regency, East Java Province, Indonesia. The results of the study are depicted in **Table 3** showing that yield, diameter and plant height were significantly enhanced in PlantGuard treatment groups compared to the control group.

**Table 3:** Effect of PlantGuard (PG) on shallot yield and growth parameters in field trials conducted in Indonesia.

Treatment	N Data	Total yield (kg)	Plant height (cm)	Diameter (cm)	Bulb count
Control	3	16.1 ± 0.08	35.4 ± 0.05	2.9 ± 0.12	4.5 ± 0.40
Competitor 2 mL/L	3	17.8 ± 0.25	36.1 ± 0.23	3.4 ± 0.14	4.9 ± 0.71
PG 0.25 mL/L	3	17.1 ± 0.09	36.2 ± 0.37	3.3 ± 0.21	4.7 ± 1.06
PG 0.3 mL/L	3	18.6 ± 0.26*	37.5 ± 0.17	3.7 ± 0.06**	4.7 ± 0.36
PG 0.6 mL/L	3	18.2 ± 0.16	37.2 ± 0.24**	3.5 ± 0.16	4.6 ± 0.47
p-value		*≤ 0.05	**≤ 0.01	**≤ 0.01	

Shallot plants were treated with different concentrations of PlantGuard (PG) and compared with untreated controls and a competitor product (2 mL/L). Data are shown as mean ± standard error (n = 3). PG treatments significantly improved several agronomic traits. Total yield was significantly increased in the PG 0.3 mL/L group (18.6 ± 0.26 kg) compared with the control (16.1 ± 0.08 kg, p = 0.02). Plant height was also significantly enhanced under PG application, with the highest values observed at 0.3 mL/L (37.5 ± 0.17 cm) and 0.6 mL/L (37.2 ± 0.24 cm), compared with control plants (35.4 ± 0.05 cm, p **≤** 0.01). Bulb diameter was significantly increased in PG treatments, particularly at 0.3 mL/L (3.7 ± 0.06 cm), relative to control (2.9 ± 0.12 cm, p **≤** 0.01). Bulb count per plant showed no significant differences between treatments. These findings indicate that PG application enhances yield and growth traits in shallots, with optimal effects observed at 0.3 mL/L. Statistical significance was defined as *p **≤** 0.05 and **p **≤** 0.01.

**Supplementary Table 1** presents rice yield data from a controlled study comparing PlantGuard (PG) at three concentrations (0.25, 0.3, and 0.6 mL/L) against a competitor product (2 mL/L) and an untreated control, each with 24 different lots. The control group shows a baseline total yield of 15.17 ± 0.40 kg. All treatments resulted in increased yields relative to the control, with percentage improvements ranging from 15.9% (PG 0.25 mL/L) to 20.3% (PG 0.6 mL/L). The competitor achieved an 18.4% increase (17.96 ± 0.51 kg), which is comparable to PG at 0.3 mL/L (17.96 ± 0.46 kg). Among the PlantGuard concentrations evaluated, 0.6 mL/L produced the greatest yield (18.25 ± 0.50 kg). Statistical analysis showed that several treatment groups differed significantly from the control, with some comparisons reaching p ≤ 0.01 (**Supplementary Table 1**).

Furthermore, PlantGuard was evaluated as a foliar treatment of kale (*Brassica oleracea* var. *sabellica*) cultivated under hydroponic conditions. After weekly foliage spray application of PlantGuard at 20ppm, the leaves of treatment and control groups were harvested and the weight determined. The results show that treatment with PlantGuard nearly tripled the kale yield compared to the control group with a percentage yield increase of 194.2% (**Figures 1A, B**). The yield increase was accompanied by a strong increase in leaf size. To extend our exploratory work beyond kale, tomato was used as a model fruiting crop for subsequent molecular analyses of PlantGuard-associated responses. **Figures 1C, D** shows the growth-promoting properties of PlantGuard when applied to tomatoes grown under controlled hydroponics conditions.

**Figure 1:**
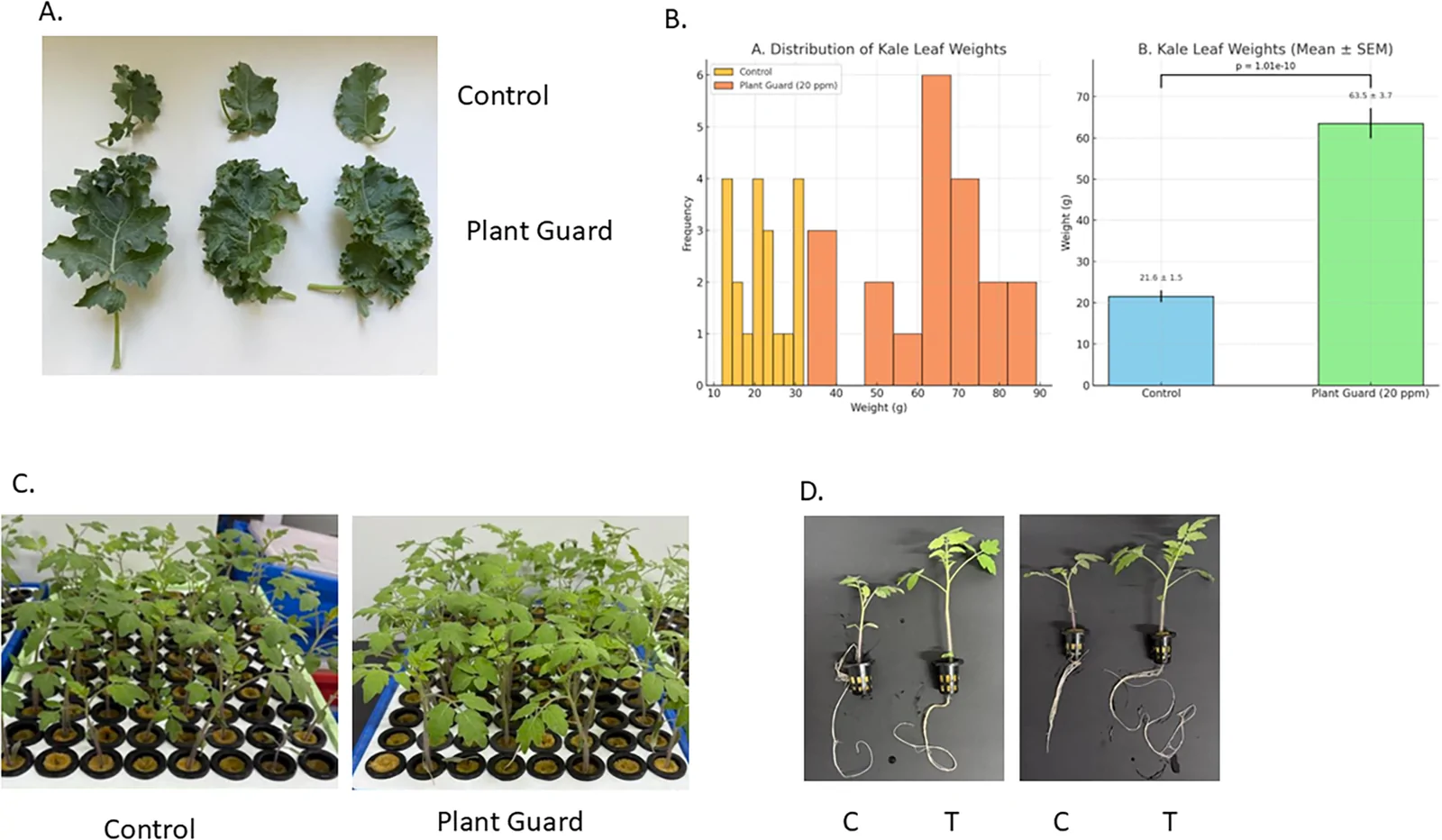
Growth stimulatory efficacy of PlantGuard in kale and tomato plants. Kale plants growing in a hydroponics system were treated weekly via foliar spray application with a 20ppm dilution of PlantGuard oil from the seedling stage up to point of harvest (6 weeks). Representative leaves from control and treatment groups were photographed **(A)** and all leaves from 20 plants per group were harvested and the weight (in g) determined. The distribution of kale leaf weights was plotted in histogram form and means ± SEM determined followed by an ANOVA statistical analysis to determine significance **(B)**. Tomato plants growing under hydroponics conditions were photographed at different stages as from control and PlantGuard treated groups **(C)**, with a closer display of two representative plants from each group **(D)**.

To determine whether the observed growth response could be attributed to the complete PlantGuard formulation rather than to the Tween 80-containing carrier alone, an additional hydroponic experiment was performed with tomato. Plants treated with PlantGuard (0.6 mL/L) showed a mean stem length of 12.17 ± 0.54 cm compared with 8.65 ± 0.80 cm in plants receiving the matched Tween 80/water carrier control, corresponding to a 40.7% increase. The difference was statistically significant (Welch’s unpaired two-tailed t-test, p = 0.0057; n = 6 biological replicates per group; **Supplementary Figure 4**). These results indicate that the increased stem growth observed under these experimental conditions was not reproduced by the Tween 80-containing carrier alone.

### 3.2 Transcriptomic responses associated with PlantGuard treatment

RNA-seq has been widely applied in tomato to characterize transcriptional responses associated with development, environmental stress, and elicitor or biostimulant treatments (de Oliveira Sátiro et al., 2026; Zhao et al., 2022). Transcriptomic profiling of 5 untreated control and 5 PlantGuard-treated 14-day-old tomato seedlings revealed treatment-associated differences in gene expression. Principal component analysis (PCA) showed separation between most control and PlantGuard-treated samples, while box plots showed comparable gene-expression distributions across samples (**Supplementary Figure 1A**).

The volcano plot revealed a large number of differentially expressed genes (DEGs) in PlantGuard-treated tomato seedlings compared with controls (**Supplementary Figure 1B**).

Differential-expression analysis identified 2,486 significant DEGs between PlantGuard-treated and control plants (|log_2_FC| ≥ 1, padj ≤ 0.05), comprising 1,315 upregulated and 1,171 downregulated genes. Representative significant DEGs included photosynthesis-related PSBS (Solyc06g060340.3; log_2_FC = 2.12, padj = 0.0042) and psaD (Solyc06g054260.1; log_2_FC = 1.65, padj = 0.0033), the ammonium transporter AMT1-3 (Solyc03g045070.1; log_2_FC = 1.92, padj = 0.0227), a jasmonic-acid-associated gene (Solyc12g013620.2; log_2_FC = 1.75, padj = 0.0111), and an auxin response factor (Solyc04g081235.1; log_2_FC = −1.37, padj = 0.0248). Additional representative DEGs related to photosynthesis, hormone responses and stress/defense are provided in **Supplementary Table 2**.

Gene Ontology (GO) and Kyoto Encyclopedia of Genes and Genomes (KEGG) enrichment analyses were used to characterize the biological functions and pathways associated with the differentially expressed genes (Ashburner et al., 2000; The Gene Ontology Consortium, 2021; Kanehisa and Goto, 2000; Kanehisa et al., 2022). GO and KEGG enrichment analyses identified significant enrichment of photosynthesis-related processes in PlantGuard-treated tomato seedlings (**Figures 2A, B**). Enriched GO terms included ‘photosynthesis,’ ‘light harvesting,’ and ‘photosynthetic light reaction,’ while KEGG analysis identified enrichment of pathways including ‘photosynthesis,’ ‘photosynthesis – antenna proteins,’ and ‘carbon fixation in photosynthetic organisms.’ These findings demonstrate treatment-associated transcriptional changes involving photosynthesis and carbon fixation. Specifically, numerous differentially expressed genes were associated with photosynthesis, secondary metabolism, and stress-response signaling (**Supplementary Table 2**). Collectively, these transcriptional changes are consistent with PlantGuard-associated effects on processes related to photosynthesis, secondary metabolism, and stress responses.

**Figure 2:**
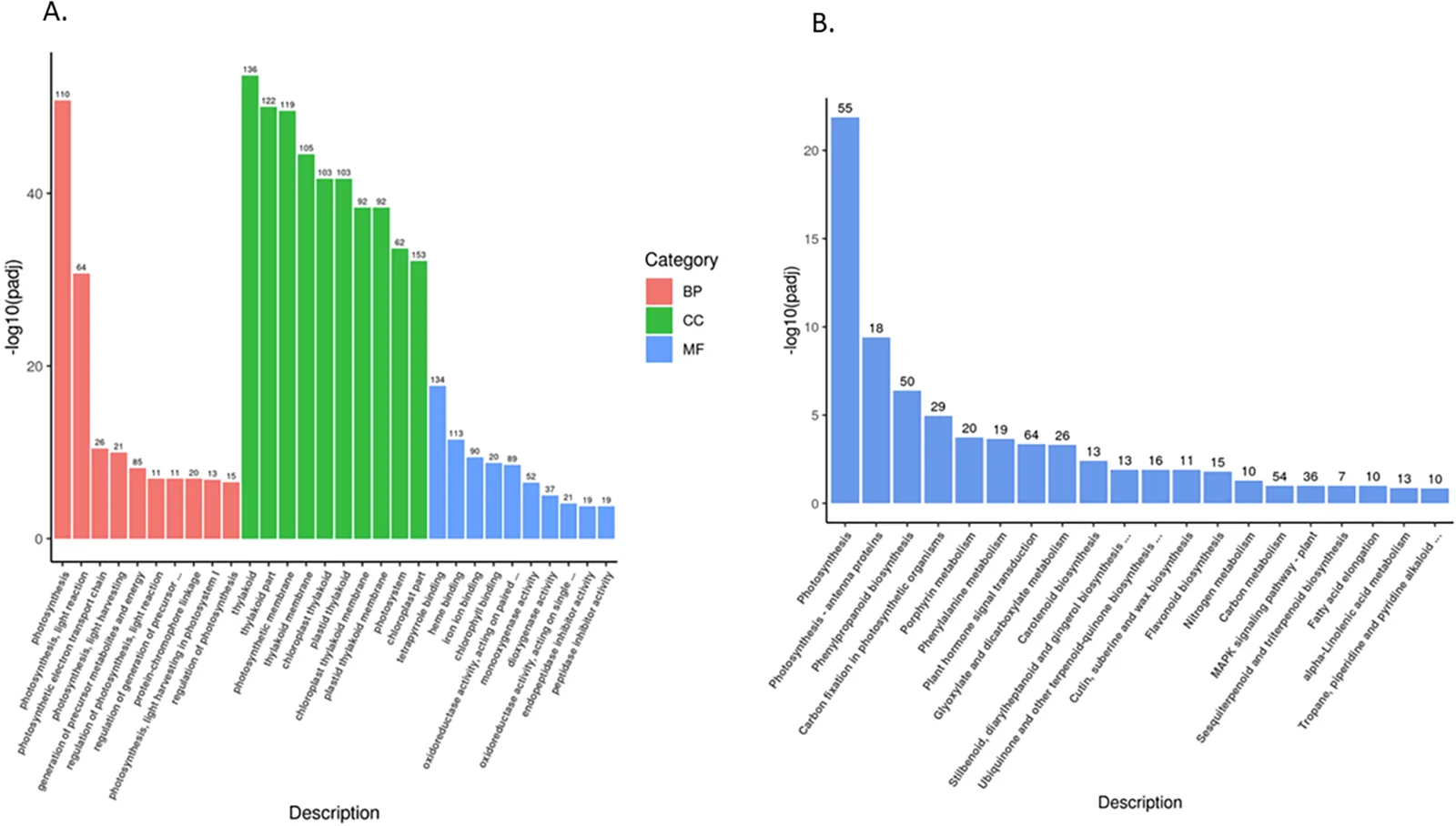
Gene Ontology (GO) and KEGG pathway enrichment analysis of differentially expressed genes in PlantGuard–treated tomato plants. **(A)** GO enrichment analysis showing significantly enriched terms in three categories: Biological Process (BP), Cellular Component (CC), and Molecular Function (MF). Highly enriched terms included photosynthesis-related processes, chloroplast and thylakoid localization, and oxidoreductase activity. **(B)** KEGG pathway enrichment analysis of differentially expressed genes, with top enriched pathways including photosynthesis, carbon fixation, porphyrin and chlorophyll metabolism, phenylpropanoid biosynthesis, and plant hormone signal transduction. Together, these enrichment patterns indicate treatment-associated transcriptional changes involving photosynthetic, metabolic, and defense-related processes.

### 3.3 Proteomic responses associated with PlantGuard treatment

Proteomic profiling of PlantGuard-treated and control tomato samples was performed by data-independent acquisition (DIA) mass spectrometry using an Orbitrap Astral high-resolution mass spectrometer. A total of 7,991 proteins were identified and quantified across the 12 samples. Using the significance criteria defined in the Materials and Methods, 208 proteins differed significantly in abundance between PlantGuard-treated and control samples, of which 156 showed increased and 52 decreased abundance.

The distribution of differentially abundant proteins was visualized by volcano plot analysis, while hierarchical clustering showed their relative abundance patterns across samples (**Supplementary Figures 2A, B**). Subcellular localization analysis further characterized the distribution of these proteins across cellular compartments (**Supplementary Figure 2C**). GO annotation of the proteomics dataset showed that the largest Biological Process categories included oxidation–reduction processes (521 proteins), protein phosphorylation (279), general metabolic processes (275), carbohydrate metabolic processes (188), translation (187), and proteolysis (140) (**Figure 3A**). Within the Cellular Component category, prominent groups included ribosomal proteins (183), integral (175) and intrinsic (155) membrane components, nuclear proteins (106), and photosystem-associated proteins, including photosystem II (24) and the photosystem II oxygen-evolving complex (20). The largest Molecular Function categories included protein binding (612), ATP binding (505), protein kinase activity (279), nucleic acid binding (264), and oxidoreductase activity (221).

**Figure 3:**
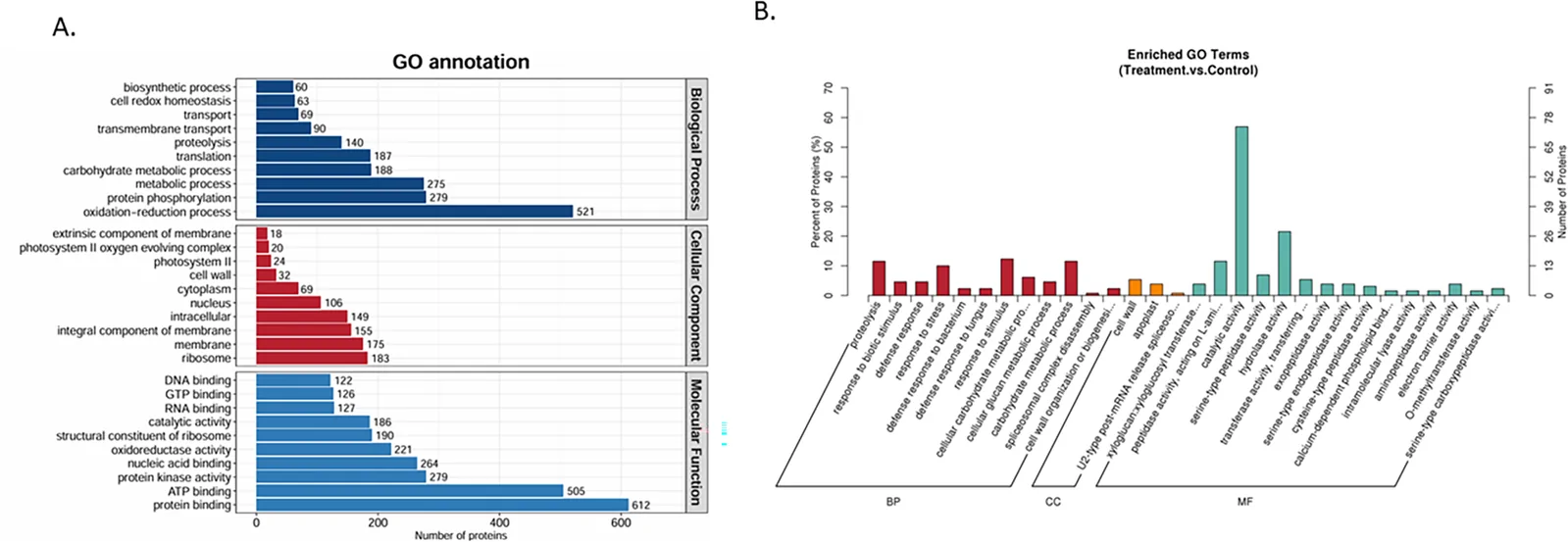
Gene Ontology (GO) annotation and enrichment analysis of differentially abundant proteins identified in PlantGuard–treated tomato plants. **(A)** GO annotation of identified proteins categorized into Biological Process (BP), Cellular Component (CC), and Molecular Function (MF). Prominent protein classes included oxidation–reduction processes, metabolic and carbohydrate metabolic processes, ribosomal components, and protein binding. **(B)** GO enrichment analysis of proteins showing significantly different abundance between PlantGuard-treated and control samples. Enriched categories included defense responses to biotic and abiotic stimuli, proteolysis, cell-wall organization, peptidase activity, and catalytic activity, indicating treatment-associated protein-abundance changes involving stress responses, structural organization, and metabolic processes.

GO enrichment analysis comparing PlantGuard-treated and control samples revealed significant overrepresentation of categories associated with defense and stress responses (**Figure 3B**). Within the Biological Process domain, enriched terms included proteolysis, defense response to biotic stimulus, and responses to stress, bacteria, and fungi. Enriched Cellular Component terms included the cell wall and apoplast, while Molecular Function terms included peptidase and hydrolase activities, particularly serine-type peptidases, as well as transferase and glycosyltransferase activities. Collectively, these enrichment patterns were associated with processes related to stress responses, protein turnover, and cell-wall organization.

Several proteins showed markedly higher abundance in the PlantGuard treatment group (**Supplementary Table 3**), including proteins associated with stress responses, hormone signaling, cell-wall organization, and photosynthetic processes. Non-specific lipid-transfer protein 1 (P27056) showed a greater than five-fold increase in abundance. Abscisic stress-ripening protein 1 (Q08655) and the ethylene-responsive late embryogenesis-like protein (P93220) also showed increased abundance, consistent with treatment-associated changes involving ABA- and ethylene-related stress responses. Proteins associated with cell-wall organization, including probable xyloglucan endotransglucosylase/hydrolase 1 (Q40144) and expansin (Q6KEZ3), were also more abundant in PlantGuard-treated samples. In addition, STAY-GREEN protein (A0A3Q7HGJ1) showed significantly increased abundance, consistent with the transcriptomic changes observed for photosynthesis-associated genes. Collectively, these protein-abundance changes were associated with stress responses, hormone-related signaling, cell-wall organization, and photosynthetic processes.

### 3.4 Metabolomic responses associated with PlantGuard treatment

Untargeted liquid chromatography–mass spectrometry (LC-MS) metabolomic profiling was performed to characterize treatment-associated changes in metabolites in PlantGuard-treated and control tomato samples. PCA and volcano plot analyses showed the overall distribution of metabolites and significantly different metabolites between PlantGuard-treated and control samples (**Supplementary Figures 3A, B**). Cluster analysis further showed treatment-associated patterns in metabolite abundance (**Supplementary Figure 3C**) Pathway enrichment analysis identified treatment-associated differences across multiple primary and secondary metabolic pathways (**Figures 4A, B**). In positive ion mode, enriched pathways included tryptophan metabolism, arginine and proline metabolism, flavonoid biosynthesis, pantothenate and CoA biosynthesis, and riboflavin metabolism. In negative ion mode, enriched pathways included the citrate cycle (TCA cycle), glyoxylate and dicarboxylate metabolism, pyruvate metabolism, flavone and flavonol biosynthesis, and porphyrin and chlorophyll metabolism. Collectively, these enrichment patterns were associated with amino acid metabolism, central carbon and energy metabolism, and secondary metabolite biosynthesis. Untargeted metabolomics identified multiple compounds that were significantly more abundant in the PlantGuard treatment group (**Supplementary Table 4**), several of which were consistent with the transcriptional and proteomic findings. Acetyl-maltose (FC ~10.3) and 2-dehydro-3-epi-20-hydroxyecdysone were among the most enriched metabolites and have been associated with plant defense-related processes. The flavonoid glycosides astragalin and isoquercetin were also elevated, consistent with the observed enrichment of flavonoid biosynthesis and changes in phenylpropanoid pathway-associated enzymes. Epoxypheophorbide a and pheophorbide A, chlorophyll catabolites, were enriched in the treatment group, alongside the increased abundance of STAY-GREEN detected in the proteomic analysis. Phosphoenolpyruvate (PEP), a central metabolite in glycolysis and precursor for secondary metabolism, was also significantly elevated. In addition, abscisic acid was detected together with increased abundance of ABA-associated proteins, including ASR and the abscisic acid receptor PYL1. Collectively, these metabolite changes were associated with defense-related secondary metabolism, chlorophyll metabolism, central carbon metabolism, and hormone-related responses.

**Figure 4:**
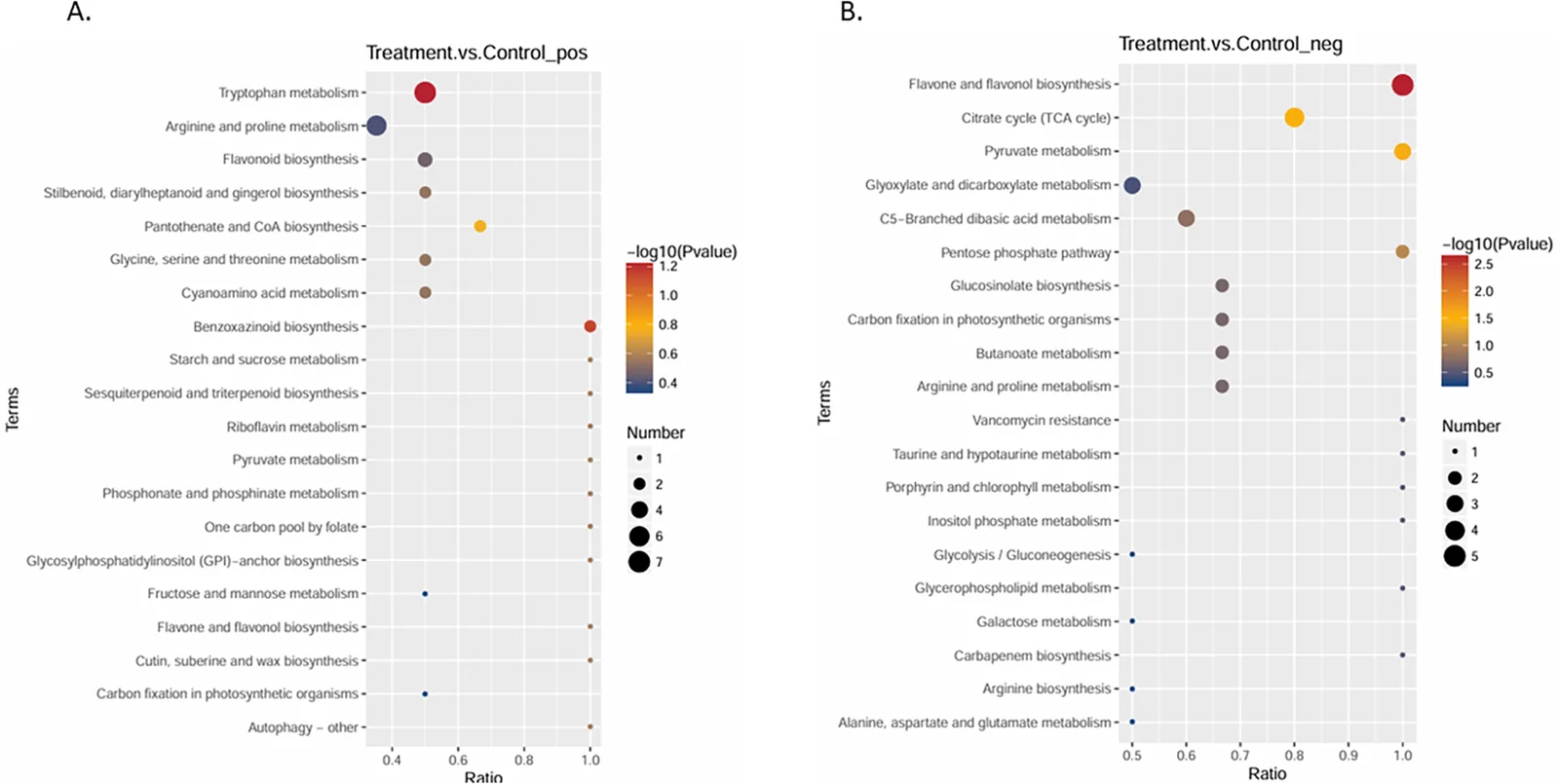
KEGG pathway enrichment of differentially accumulated metabolites in PlantGuard–treated tomato plants. **(A)** Enrichment analysis in positive ion mode identified pathways including tryptophan metabolism, arginine and proline metabolism, flavonoid biosynthesis, and pyruvate metabolism. **(B)** Enrichment analysis in negative ion mode identified pathways including flavone and flavonol biosynthesis, citrate cycle (TCA cycle), glyoxylate and dicarboxylate metabolism, and carbon fixation in photosynthetic organisms. Bubble size represents the number of mapped metabolites, while color indicates significance level (−log10 p-value).

## 4 Discussion

Field evaluations of PlantGuard in Indonesia demonstrated agronomic effects, with a 32.6% increase in tomato yield recorded in the highland agricultural region of Pangalengan District, Bandung Regency, West Java, and a 15.5% increase in shallot yield under local cultivation practices. These results were complemented by controlled hydroponic trials with kale, which showed a 194% increase in yield following foliar application of PlantGuard. Together, these field and controlled-environment studies provide evidence of PlantGuard-associated growth responses and provided the basis for subsequent integrative omics investigations aimed at characterizing molecular and metabolic changes associated with treatment.

The field trials also indicated crop-dependent differences in the apparent optimal PlantGuard application rate. In tomato, the strongest yield response was observed at 0.24 mL/L, whereas higher concentrations did not result in a corresponding further increase in yield. In contrast, the rice trials showed the strongest response at 0.6 mL/L, the highest concentration evaluated in that crop. Several factors may contribute to these differences, including crop-specific physiology, growth duration, and environmental conditions. Notably, the tomato trial was conducted under highland conditions, whereas the rice trials were performed in lowland cultivation systems. These differences may influence crop responsiveness to PlantGuard and indicate that an application rate identified for one crop or cultivation environment should not necessarily be extrapolated directly to another. The present trials were not designed to determine the physiological basis of these crop-specific dose responses, and no dedicated phytotoxicity or residue analyses were performed. Further dose-ranging studies, including intermediate concentrations and assessment of potential phytotoxicity, will therefore be required to define crop-specific effective application ranges.

To characterize PlantGuard-associated molecular responses at different developmental stages, RNA sequencing was performed in 14-day-old tomato seedlings, followed by proteomic and metabolomic profiling in 4-week-old plants. Because an emulsifier-only control was not included in the RNA-seq experiment, the observed transcriptional responses should be interpreted as being associated with the complete PlantGuard formulation and cannot be attributed specifically to its essential-oil constituents.

The seedling stage was selected for transcriptomic analysis to capture early treatment-associated gene-expression responses, while proteomic and metabolomic analyses in older plants provided complementary information on protein-abundance and metabolic changes. Together, these analyses enabled molecular responses associated with PlantGuard treatment to be examined across multiple omics layers and developmental stages.

Although targeted RT-qPCR validation was not performed, integration of the transcriptomic, proteomic, and metabolomic datasets provided complementary evidence for treatment-associated molecular responses. Concordant changes across the different omics layers were observed for processes related to photosynthesis, central metabolism, stress responses, and secondary metabolism. These integrated findings support an association between PlantGuard treatment and coordinated molecular responses across several biological processes, while functional validation will be required to establish causal relationships and specific regulatory mechanisms. The model shown in **Figure 5** summarizes these integrated observations as a proposed Photosynthesis/Precursor Hub, based on associations identified across the transcriptomic, proteomic, and metabolomic datasets.

**Figure 5:**
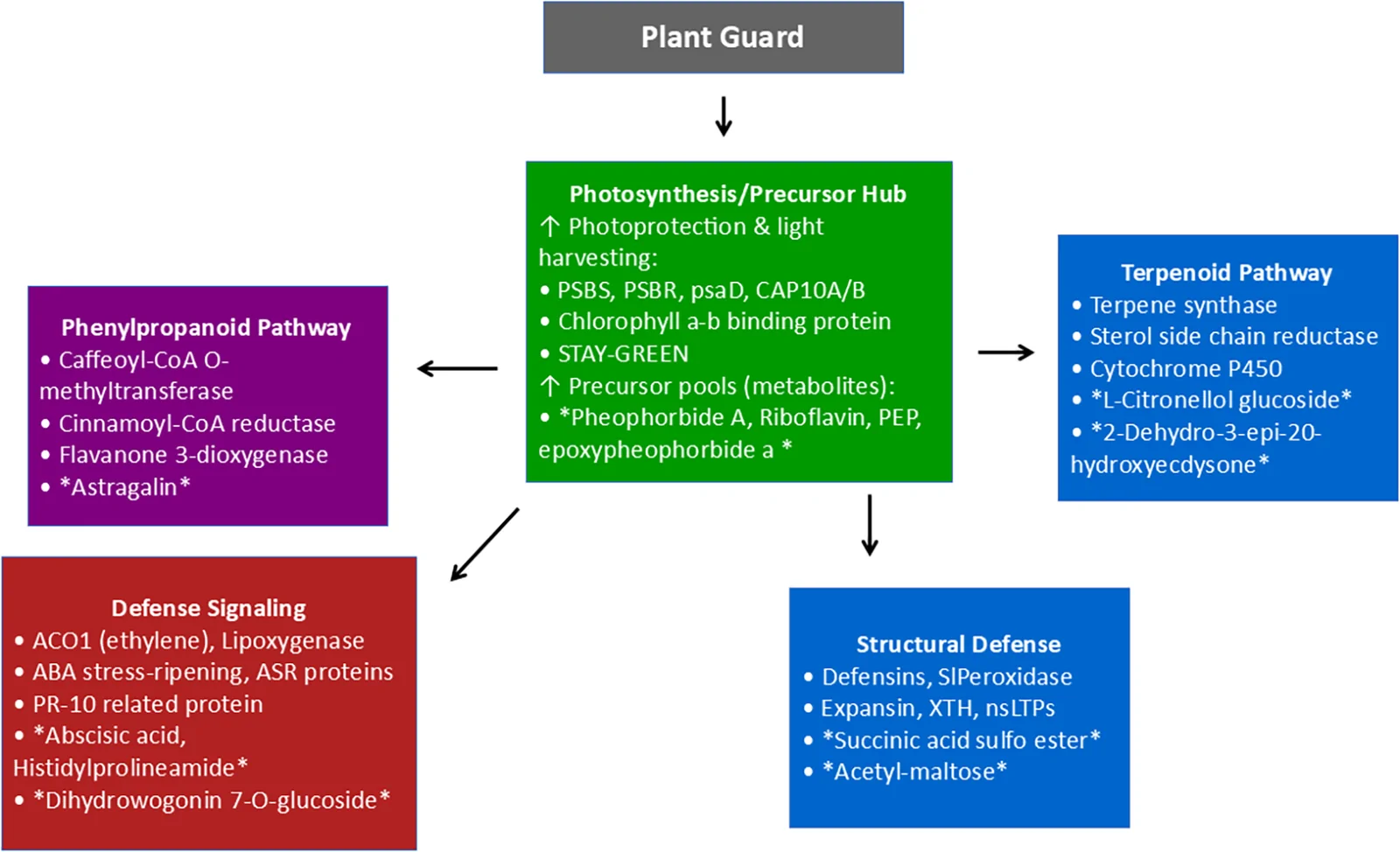
Schematic model summarizing treatment-associated changes identified by integrative multi-omics analysis of PlantGuard-treated tomato plants. The model integrates treatment-associated changes in photosynthesis-related genes and proteins (including PSBS, PSBR, psaD, CAP10A/B, chlorophyll a–b binding proteins, and STAY-GREEN) with changes in metabolites associated with photosynthetic and central metabolic processes, including pheophorbide A, riboflavin, phosphoenolpyruvate, and epoxypheophorbide a. These molecular changes were associated with phenylpropanoid and terpenoid metabolism, defense-related signaling, and structural defense processes. The schematic represents an integrative interpretation of the observed transcriptomic, proteomic, and metabolomic associations and does not imply direct functional validation of the proposed relationships.

At the transcript level, we observed significant upregulation of photosystem-associated genes including PSBS, PSBR, psaD, CAP10A, and CAP10B. These encode proteins involved in light harvesting, PSI/PSII stabilization, and non-photochemical quenching (NPQ). The increased expression of PSBS, in particular, is consistent with improved photoprotection under high light conditions, a role well established in Arabidopsis (Li et al., 2000; Külheim et al., 2002). Similarly, psaD supports PSI electron transport by stabilizing ferredoxin interactions. Effects of psaD mutations on PSI stability were reported (Ihnatowicz et al., 2004). The proteomics data further confirmed upregulation of chlorophyll a–b binding proteins and the STAY-GREEN protein, aligning with metabolite accumulation of pheophorbide A and epoxypheophorbide a, intermediates of chlorophyll turnover, and riboflavin, a cofactor implicated in redox regulation. Together, these findings indicate coordinated treatment-associated changes in molecular components related to photosynthesis, photoprotection, and chlorophyll metabolism, consistent with the proposed Photosynthesis/Precursor Hub model.

Metabolomic analysis provided further support for the proposed Photosynthesis/Precursor Hub, with increased abundance of metabolites associated with photosynthetic and central metabolic processes, including phosphoenolpyruvate (PEP), riboflavin, and chlorophyll catabolites. These changes were accompanied by treatment-associated differences in specialized metabolic pathways. Phenylpropanoid-related changes included increased abundance of caffeoyl-CoA O-methyltransferase and cinnamoyl-CoA reductase, together with accumulation of astragalin and isoquercetin, while terpenoid-related changes involved terpene synthase, sterol side chain reductase, cytochrome P450s, and metabolites such as L-citronellol glucoside and 2-dehydro-3-epi-20-hydroxyecdysone. Collectively, these observations are consistent with coordinated changes in central and specialized metabolism following PlantGuard treatment, including pathways associated with plant defense and stress responses.

Concomitantly, treatment-associated changes were observed in several defense- and stress-related pathways. The RNA-seq dataset showed upregulation of 1-aminocyclopropane-1-carboxylate oxidase 1 (ACO1), a key enzyme in ethylene biosynthesis, as well as lipoxygenase and cytochrome P450s, which are associated with jasmonate- and oxylipin-mediated defense signaling (Wasternack and Feussner, 2018). Metabolomic analysis identified increased abundance of abscisic acid and histidylprolineamide, both associated with stress-related responses, while proteomic analysis showed increased abundance of the abscisic acid receptor PYL1 and pathogenesis-related proteins. Together, these observations are consistent with PlantGuard-associated changes involving hormone-related signaling and defense-related processes; however, functional studies would be required to establish whether these changes result in defense priming.

Finally, increased abundance of defense- and cell-wall-associated proteins, including defensin-like proteins, SlPeroxidase, expansins, xyloglucan endotransglucosylases/hydrolases (XTHs), and lipid-transfer proteins (nsLTPs), was observed following PlantGuard treatment. These changes were accompanied by metabolomic detection of succinic acid sulfo esters and acetyl-maltose, which were associated with processes related to lignification and suberization. Collectively, these molecular changes are consistent with treatment-associated responses involving chemical defense and cell-wall organization, although their contribution to functional resistance against pathogens was not directly assessed in the present study.

The biological responses associated with PlantGuard treatment may also be considered in relation to its major oil constituents, several of which are well-characterized plant volatiles with reported elicitor activity. Linalool, a common oxygenated monoterpene, has been shown to induce defense gene expression and modulate jasmonic acid (JA) and ethylene (ET) signaling, while also acting as an attractant for natural enemies of herbivores (Arimura et al., 2009; Zhang et al., 2019). Eucalyptol (1,8-cineole) and α-terpineol likewise function as antimicrobial and signaling volatiles, with reported links to salicylic acid (SA)-mediated defenses and redox regulation (Maffei et al., 2011; Lee et al., 2021). γ-Terpinene belongs to a class of volatile terpenes associated with mitigation of oxidative stress and photoprotection, which is of interest in relation to the treatment-associated changes in photosystem-related genes observed in the present study (Loreto and Schnitzler, 2010). Benzenoid volatiles such as benzaldehyde and benzyl acetate are produced via phenylpropanoid metabolism and have been reported to act as antimicrobial agents and defense signals, with evidence for priming JA/ET-responsive transcription factors (Dudareva et al., 2013; Turlings and Erb, 2018). Plant VOCs have also been reported to activate Ca²^+^ influx, ROS bursts, and MAP kinase cascades that converge on ABA, JA, and ET signaling pathways (Maffei et al., 2011). These previously reported activities provide a plausible biological context for the treatment-associated molecular changes observed in the present study. However, the contribution of individual PlantGuard constituents to these responses and potential synergistic interactions among the constituents were not directly investigated.

In summary, the integrated omics data support a proposed model in which PlantGuard treatment is associated with coordinated changes in photosynthesis-related processes, central metabolism, secondary metabolism, hormone- and redox-related signaling, and cell-wall organization. These observations form the basis of the proposed Photosynthesis/Precursor Hub shown in **Figure 5**, which provides an integrative framework for interpreting the transcriptomic, proteomic, and metabolomic responses observed in this study. While the concordance across multiple omics layers supports the biological relevance of these associations, the proposed relationships between these processes remain hypotheses that will require targeted functional validation.

## 5 Conclusions

The present study demonstrates that PlantGuard, an essential-oil-based biostimulant, is associated with measurable molecular and agronomic responses across multiple experimental systems. Integrative transcriptomic, proteomic, and metabolomic analyses identified coordinated treatment-associated changes in processes related to photosynthesis, central and secondary metabolism, stress responses, and hormone-related signaling. These molecular observations were complemented by hydroponic and field trial data showing improvements in plant growth parameters and crop yield across tomato, shallot, rice, and kale cultivation systems. The convergence of multi-omics and agronomic observations provides a systems-level framework for understanding the biological responses associated with PlantGuard treatment, while targeted functional studies will be required to establish the causal mechanisms underlying these responses. Collectively, the findings support the potential of PlantGuard as an essential-oil-based biostimulant for sustainable crop production.

## Data Availability

The datasets presented in this study can be found in online repositories. The names of the repository/repositories and accession number(s) can be found below: https://www.ncbi.nlm.nih.gov/, GSE336220.
