# Supplementary material for: Integrative omics and field studies reveal the efficacy of PlantGuard, a next-generation biostimulant
Source: Front Plant Sci. 2026 Sep 30;17:1918122. doi: 10.3389/fpls.2026.1918122 (PMC13661932; doi:10.3389/fpls.2026.1918122)
Supplement: Supplementary file 1 [file Table1.docx]

| **Treatment** | **N Data** | **Yield (%)** | **Total Yield (kg)** |
| --- | --- | --- | --- |
| Control | 24 | 0 | 15.17 ± 0.40 |
| Competitor 2 mL/L | 24 | 18.4 | 17.96 ± 0.51* |
| PG 0.25 mL/L | 24 | 15.9 | 17.58 ± 0.49* |
| PG 0.3 mL/L | 24 | 18.4 | 17.96 ± 0.46* |
| PG 0.6 mL/L | 24 | 20.3 | 18.25 ± 0.50** |
| **p-value** | |  | ***<0.05 **<0.01** |
