# Supplementary material for: Integrative omics and field studies reveal the efficacy of PlantGuard, a next-generation biostimulant
Source: Front Plant Sci. 2026 Sep 30;17:1918122. doi: 10.3389/fpls.2026.1918122 (PMC13661932; doi:10.3389/fpls.2026.1918122)
Supplement: Supplementary file 2 [file Table2.docx]

| **Gene ID** | **Gene Description** | **Function** | **Treatment vs Control (log2FC)** | **p-value** |
| --- | --- | --- | --- | --- |
| Solyc05g050350.2 | Cyclic nucleotide-gated channel | Resistance in tomato via Ca2+ signaling | 9.039119494 | 1.15E-11 |
| Solyc07g039570.3 | CsbHLH041 | Increased salt and ABA tolerance | 7.348307288 | 3.43E-05 |
| Solyc02g067600.1 | Dehydrin family protein | Promoting stress tolerance | 7.278938511 | 6.99E-06 |
| Solyc01g079890.3 | Nodulin26-like intrinsic protein 71 | Aquaporin, counteracting water stress) | 7.11712631 | 8.32E-07 |
| Solyc01g057080.1 | Ethylene-responsive transcription factor | Fruit riping process | 6.436536958 | 2.86E-05 |
| Solyc05g052420.2 | Abscisic acid receptor | Counteracting effects of bouts of severe drought | 6.350463965 | 4.03E-05 |
| Solyc07g049530.3 | 1-aminocyclopropane-1-carboxylate oxidase 1 | Makes the plant hormone Ethylene | 6.315800023 | 3.42E-09 |
| Solyc01g014140.3 | Hcr9-OR3A | Resistance against Cladosporium fulvum | 5.420827569 | 7.35E-07 |
| Solyc01g105890.3 | Monoterpene synthase 1 | Catalyzes the formation of Linalool | 4.016559357 | 5.97E-11 |
| Solyc07g009030.3 | Defensin-like protein | Antifungal, antibacterial and anti-insect activity | 4.002337859 | 0.000118 |
| Solyc07g005370.3 | (PR)-10-related norcoclaurine synthase-like | Resistance to biotic stress | 3.472107456 | 0.000113 |
| Solyc07g008020.3 | AUXIN RESPONSE FACTOR 5 | Regulates fruit set and development | 2.399521148 | 0.001096 |
| Solyc09g064500.3 | Photosystem II reaction center Psb28 protein | Photosynthesis | 2.084245936 | 0.000642 |
| Solyc07g066310.3 | Photosystem II 10 kDa polypeptide | Photosynthesis, Photosystem II 10 kDa polypeptide | 2.026425173 | 0.001424 |
| Solyc06g069730.3 | Chlorophyll a-b binding protein | Photosynthesis | 2.002022225 | 0.000478 |
| Solyc12g009200.2 | Chlorophyll a-b binding protein | Photosynthesis | 1.978667897 | 1.09E-05 |
| Solyc12g006140.2 | Chlorophyll a-b binding protein | Photosynthesis | 1.935928795 | 0.000897 |
| Solyc06g060340.3 | PSBS | Photosynthesis, Photosystem II 22 kDa protein | 2.12097952 | 4.81E-05 |
| Solyc07g066310.3 | PSBR | Photosynthesis, Photosystem II 10 kDa polypeptide | 2.12097952 | 4.81E-05 |
| Solyc06g054260.1 | psaD | Photosynthesis, Photosystem I reaction center subunit II | 2.026425173 | 0.001424 |
| Solyc01g105050.3 | CAP10B | Photosynthesis, Chlorophyll a-b binding protein CP24 10B | 1.650626977 | 3.13E-05 |
| Solyc01g105030.3 | CAP10A | Photosynthesis, Chlorophyll a-b binding protein CP24 10A | 1.338331097 | 0.000226 |
| Solyc09g064400.2 | psaJ | Photosynthesis, Photosystem I reaction center subunit IX | 1.24620659 | 0.000571 |
| Solyc01g102770.1 | psbZ | Photosynthesis, Photosystem II reaction centre protein Z | 1.22184003 | 0.278547 |
