# Supplementary material for: Integrative omics and field studies reveal the efficacy of PlantGuard, a next-generation biostimulant
Source: Front Plant Sci. 2026 Sep 30;17:1918122. doi: 10.3389/fpls.2026.1918122 (PMC13661932; doi:10.3389/fpls.2026.1918122)
Supplement: Supplementary file 3 [file Table3.docx]

| **Protein** | **Description** | **Function** | **Treatment.vs.Control FC** | **Treatment.vs.Control p-value** |
| --- | --- | --- | --- | --- |
| P27056 | Non-specific lipid-transfer protein 1 | Stress response to drought | 5.419871027 | 0.011953805 |
| Q40144 | Probable xyloglucan endotransglucosylase/hydrolase 1 | Cell wall protein; Seedling growth | 4.490879934 | 0.006688702 |
| Q08655 | Abscisic stress-ripening protein 1 | Stress response to drought | 4.423470721 | 0.006405036 |
| P93224 | Non-specific lipid-transfer protein 2 | Stress response to drought | 4.152741978 | 0.021347354 |
| P93220 | Ethylene-responsive late embryogenesis-like protein | Stress response to drought | 3.538120551 | 0.000201814 |
| A0A3Q7G376 | Alpha-galactosidase | Cell wall protein; Seedling growth | 3.286085437 | 0.000186183 |
| A0A3Q7F6V7 | Flavanone 3-dioxygenase | Flavonoid biosynthesis, stress tolerance | 3.203576123 | 0.001027871 |
| A0A3Q7FI29 | Xyloglucan endotransglucosylase/hydrolase | Tomato softening | 3.141188461 | 0.011483309 |
| Q4A1N1 | Non-specific lipid-transfer protein | Defence against pathogens | 2.906405995 | 0.009339761 |
| Q6KEZ3 | Expansin | Fruit ripening | 2.877013657 | 0.005250941 |
| Q2QJT5 | ASR4 | Stress response to drought | 2.87445857 | 0.002454111 |
| Q5NE20 | Carbonic anhydrase | Plant growth and stress response | 2.695795087 | 0.001041316 |
| Q9FZ05 | Xyloglucan endotransglucosylase/hydrolase | Cell wall expansion | 2.591171083 | 0.049046478 |
| A0A0H5BXV2 | Non-specific lipid-transfer protein (Fragment) | Adaptation to environmental stresses | 2.447814946 | 0.020367943 |
| K4CVP2 | caffeoyl-CoA O-methyltransferase | Plant color, growth and development | 2.439594795 | 0.001126063 |
| Q4U1I5 | Cinnamoyl-CoA reductase | Cell wall integrity, plant growth | 2.412681403 | 0.008182334 |
| P15003 | Suberization-associated anionic peroxidase 1 | Defence against fungal pathogens | 2.356980836 | 0.001330525 |
| A0A3Q7HGJ1 | Protein STAY-GREEN | Regulator of chlorophyll breakdown | 2.340303579 | 0.028835184 |
| Q40131 | 2-oxoglutarate-dependent dioxygenase 2 | Conversion of cholesterol to a-tomatine | 2.336572749 | 0.003135003 |
| A0A0C6G3Q8 | Sterol side chain reductase | Plant growth and development | 2.245154619 | 0.039620351 |
