# Supplementary material for: Integrative omics and field studies reveal the efficacy of PlantGuard, a next-generation biostimulant
Source: Front Plant Sci. 2026 Sep 30;17:1918122. doi: 10.3389/fpls.2026.1918122 (PMC13661932; doi:10.3389/fpls.2026.1918122)
Supplement: Supplementary file 4 [file Table4.docx]

|  | **Ion Mode** | **FC** | **P-value** | **Functions** |
| --- | --- | --- | --- | --- |
| Acetyl-maltose | N | 10.31658 | 0.004898 | Resistance against insects |
| (-)-Glyceollin I | N | 9.082674 | 0.000214 | Functions in plant defense especially fungal infections |
| Lotusanine B | P | 8.513816 | 0.001418 | Unknown function in tomatoes |
| 2-Dehydro-3-epi-20-hydroxyecdysone | N | 6.812913 | 0.010616 | Defense against insects, also in tomatoes |
| Balanophonin 4-O-beta-D-glucopyranoside | P | 6.267287 | 0.000524 | Betalain synthesis, red color in tomatoes |
| (3R)-3,9-dihydroxynonanoic acid | N | 5.764892 | 0.000318 | Plant metabolism, energy production |
| L-Citronellol glucoside | N | 5.340024 | 9.97E-05 | Defense against insects, also in tomatoes |
| Histidylprolineamide | P | 5.331057 | 0.00481 | Stress response to drought |
| Phosphoenolpyruvic acid | N | 4.713977 | 0.009489 | Growth and development, energy metabolism |
| Astragalin | N | 4.621615 | 0.022344 | Defense mechanisms, role in plant health |
| Epoxypheophorbide a | P | 4.578273 | 2.28E-06 | Chlorophyll catabolism |
| Glabridin | P | 4.495611 | 0.000424 | Biostimulant effect, normally only found in licorice plants |
| Glucose lactate ketone | N | 4.478891 | 0.01154 | Defense mechanisms, role in plant health; chlorophyll metabolism |
| Isoquercetin | N | 4.444836 | 0.015118 | Antioxidant properties |
| Methyl octanoate | P | 4.281556 | 0.000648 | Could promote plant and root growth |
| Dihydrowogonin 7-O-glucoside | N | 4.229007 | 0.011734 | Defense mechanisms against pathogens and environmental stress |
| Pheophorbide A | P | 3.974827 | 6.04E-05 | Chlorophyll catabolism |
| UDP-4-dehydro-6-deoxy-D-glucose | N | 3.925873 | 0.000185 | Fruit development and ripening |
| D-Glucuronic acid | N | 3.806958 | 0.029334 | Plant growth, stress tolerance, and fruit quality. |
| 4-p-Coumaroylquinic acid | P | 3.778473 | 0.003815 | Antimicrobial and antioxidant |
| Succinic acid, sulfo-, 1,4-dioctyl ester | P | 3.282346 | 0.001828 | Promoting plant growth |
| Riboflavin | P | 2.08851 | 3.24E-05 | Defense against pathogens, B vitamin |
| Abscisic Acid | P | 1.578431 | 0.000353 | Plant hormone |
