# Supplementary material for: Integrative omics and field studies reveal the efficacy of PlantGuard, a next-generation biostimulant
Source: Front Plant Sci. 2026 Sep 30;17:1918122. doi: 10.3389/fpls.2026.1918122 (PMC13661932; doi:10.3389/fpls.2026.1918122)
Supplement: Supplementary file 5 [file Table5.docx]

Supplementary Material

**Supplementary Fig. 1**

**Quality control and differential expression overview of RNA-sequencing data.**

(A) Boxplot of normalized gene expression values (log₂[FPKM+1]) across all control (C1–C5) and PlantGuard–treated (T1–T5) tomato samples. The uniform distribution of expression levels across replicates indicates high data quality and consistency.

(B) Volcano plot showing differentially expressed genes (DEGs) between PlantGuard–treated and control groups. A total of 1,924 genes were significantly upregulated and 1,725 genes were downregulated (adjusted p < 0.05, |log₂ fold change| > 1), while 20,412 genes showed no significant change. These results confirm robust transcriptional reprogramming induced by PlantGuard treatment.

**Supplementary Fig. 2**

**Differential protein expression and subcellular localization analysis of PlantGuard–treated tomato plants.**

In (A), a Volcano plot of protein abundance is shown, comparing PlantGuard treatment with control plants. Red dots represent significantly upregulated proteins, blue dots indicate downregulated proteins, and grey dots correspond to proteins with no significant change. (B) Heatmap of differentially abundant proteins showing clear clustering of treatment versus control samples, with consistent expression patterns across biological replicates. Red indicates higher expression and blue indicates lower expression. (C) Subcellular localization analysis of identified proteins, showing the majority localized to the cytoplasm (19.02%), nucleus (15.70%), and chloroplast (15.13%), followed by proteins associated with the cell membrane, mitochondria, vacuole, and other compartments. This distribution reflects the broad impact of PlantGuard treatment on proteins involved in both primary metabolism and defence-related processes.

**Supplementary Fig. 3**

**Differential metabolite profiling of PlantGuard–treated tomato plants.**

(A) Principal component analysis (PCA) score plot showing clear separation between control (red) and PlantGuard–treated (blue) samples, indicating distinct metabolomic profiles induced by treatment. PC1 and PC2 explain 31.41% and 23.34% of the total variance, respectively. (B) Volcano plot of differentially accumulated metabolites between treatment and control groups. A total of 362 metabolites were significantly upregulated (red) and 240 metabolites were downregulated (blue), while 1,314 metabolites showed no significant change (grey). Bubble size represents the variable importance in projection (VIP) score. (C) Heatmap of differentially accumulated metabolites, clustered by treatment versus control. Distinct expression patterns highlight consistent metabolic reprogramming across replicates, with treatment samples showing coordinated shifts in metabolite abundance.

**Supplementary Table 1**

Effect of PlantGuard (PG) treatments on rice yield under field conditions. Total yield (kg) is presented as mean ± SEM based on n = 24 independent measurements per treatment. Yield (%) represents the relative increase compared to the untreated control. PG was applied at three concentrations (0.25, 0.3, and 0.6 mL/L) and compared to a commercial competitor (2 mL/L). Statistical significance was determined relative to the control group and between indicated treatment groups using one-way ANOVA followed by post hoc multiple comparison testing. Asterisks denote levels of significance (p < 0.05; p < 0.01), as indicated by brackets.

**Supplementary Table 2**

**Selected genes significantly upregulated in tomato seedlings treated with PlantGuard identified by RNA-sequencing.**

RNA-sequencing of 14-day-old tomato seedlings grown in soil revealed a range of stress-responsive and photosynthesis-related genes with significant differential expression in the PlantGuard treatment group compared to controls. Several stress- and defence-associated genes were strongly induced, including a cyclic nucleotide-gated channel (log2FC = 9.04, p = 1.15E-11), CsbHLH041 (7.35, p = 3.43E-05), a dehydrin family protein (7.28, p = 6.99E-06), and ACO1, encoding 1-aminocyclopropane-1-carboxylate oxidase (6.32, p = 3.42E-09), which is central to ethylene biosynthesis. Defence-related genes such as defensin-like proteins, PR-10 family members, and the resistance gene Hcr9-OR3A were also upregulated. At the same time, multiple photosynthesis-related genes, including PSBS, PSBR, psaD, chlorophyll a–b binding proteins, and photosystem II subunits, showed elevated expression levels, indicating enhanced light harvesting and photoprotection. Collectively, these results suggest that PlantGuard promotes a transcriptional shift toward both improved stress tolerance and strengthened photosynthetic performance.

**Supplementary Table 3**

**Proteins showing abundant expression in tomato plants treated with PlantGuard identified by DIA-based proteomics.**

Data-independent acquisition (DIA) proteomics of four-week-old hydroponically grown tomato plants revealed several proteins with significantly higher abundance in the PlantGuard treatment compared to controls. Strong upregulation was observed for stress- and drought-responsive proteins, including non-specific lipid-transfer proteins (e.g., P27056, FC = 5.42, p = 0.012), abscisic stress-ripening protein 1 (Q08655, FC = 4.42, p = 0.006), and the ethylene-responsive late embryogenesis-like protein (P93220, FC = 3.54, p = 0.0002). Cell wall–related proteins such as probable xyloglucan endotransglucosylase/hydrolase (Q40144, FC = 4.49, p = 0.007) and expansin (Q6KEZ3, FC = 2.88, p = 0.005) were also enriched, suggesting enhanced structural remodelling and growth. Additionally, proteins associated with secondary metabolism and plant defence, including flavanone 3-dioxygenase (A0A3Q7F6V7, FC = 3.20, p = 0.001), caffeoyl-CoA O-methyltransferase (K4CVP2, FC = 2.44, p = 0.001), and cinnamoyl-CoA reductase (Q4U1I5, FC = 2.41, p = 0.009), were significantly upregulated. Importantly, Protein STAY-GREEN (A0A3Q7HGJ1, FC = 2.34, p = 0.029) was also elevated, linking proteomic shifts to sustained photosynthetic activity and delayed chlorophyll degradation. These findings highlight PlantGuard’s capacity to induce both stress adaptation and growth-promoting pathways.

**Supplementary Table 4**

**Differentially enriched metabolites in tomato plants treated with PlantGuard.**

Untargeted LC–MS metabolomics revealed a range of metabolites with significantly higher abundance in the treatment group compared to controls. Notably, several compounds were associated with plant defence responses, including acetyl-maltose (FC = 10.32, p = 0.0049), (−)-glyceollin I (FC = 9.08, p = 0.0002), and 2-dehydro-3-epi-20-hydroxyecdysone (FC = 6.81, p = 0.0106), which are known to contribute to insect resistance and fungal defence. Secondary metabolites such as astragalin (FC = 4.62, p = 0.022) and glabridin (FC = 4.50, p = 0.0004) highlighted enhanced antioxidant and biostimulant properties, while isoquercetin (FC = 4.44, p = 0.015) and 4-p-coumaroylquinic acid (FC = 3.78, p = 0.0038) reflected activation of phenylpropanoid pathways. Several metabolites were linked to chlorophyll metabolism and stress signalling, including pheophorbide A (FC = 3.97, p = 6.0 × 10⁻⁵), epoxy-pheophorbide a (FC = 4.58, p = 2.3 × 10⁻⁶), and abscisic acid (FC = 1.58, p = 0.0004). Importantly, histidylprolineamide (FC = 5.33, p = 0.0048) and glucose lactate ketone (FC = 4.48, p = 0.012) were associated with drought tolerance and metabolic adaptation. Together, these results indicate that PlantGuard induces broad reprogramming of metabolic pathways, promoting defence, growth, and stress resilience.
